# The incidence and risk factors for allogenic blood transfusion in total knee and hip arthroplasty

**DOI:** 10.1186/s13018-019-1329-0

**Published:** 2019-08-28

**Authors:** Kai Song, Pin Pan, Yao Yao, Tao Jiang, Qing Jiang

**Affiliations:** 1 Department of Sports Medicine and Adult Reconstructive Surgery, Drum Tower Hospital, School of Medicine, Nanjing University, 321 Zhongshan Road, Nanjing, 210008 Jiangsu People’s Republic of China; 2 Laboratory for Bone and Joint Disease, Model Animal Research Center (MARC), Nanjing University, Nanjing, 210093 Jiangsu People’s Republic of China

**Keywords:** Total knee arthroplasty, Total hip arthroplasty, Allogenic blood transfusion, Preoperative hemoglobin level

## Abstract

**Background:**

Excessive blood loss in total joint arthroplasty (TJA) usually leads to an allogenic blood transfusion, which may cause adverse outcomes, prolonged length of hospitalization, and increased costs. The purpose of this study was to determine the incidence and risk factors for intraoperative and postoperative allogenic transfusion in patients undergoing primary unilateral total knee and hip arthroplasty (TKA and THA).

**Methods:**

We conducted a retrospective study and enrolled consecutive patients undergoing primary unilateral TKA and THA at our institution between January 2010 and July 2014 (*n* = 1534). Information about allogenic transfusion was collected from medical records to determine the incidence. We performed univariate analysis and multivariate logistic regression analysis to identify the independent risk factors.

**Results:**

Total, intraoperative, and postoperative transfusion rates were 17.9%, 7.9%, and 11.3%, respectively. The preoperative lower level of hemoglobin (Hb) (*P* < 0.001) and increased amount of intraoperative blood loss (*P* < 0.001) were independently associated with transfusion in TKA. The independent risk factors for transfusion in THA were female (*P* = 0.023), preoperative lower Hb level (*P* < 0.001), prolonged operation time (*P* < 0.001), and increased intraoperative blood loss (*P* < 0.001).

**Conclusions:**

Given the high prevalence and potential risk of transfusion in TJA, interventions for identified risk factors should be used during the perioperative period.

## Background

Total joint arthroplasty (TJA), representing an effective procedure in the treatment of various joint pathologies, is usually associated with substantial blood loss, which increases allogenic blood transfusion requirements during the perioperative period. Previous studies have proved that blood transfusion increased the risk of surgical-site infection, major complications, longer length of hospitalization, and even mortality in total knee and hip arthroplasty (TKA and THA) [1–3]. Additionally, transfusion causes higher total hospitalization cost and more medical resource use [4].

Therefore, perioperative blood management to avoid allogenic transfusion in TJA has gained increasing attention. Determining the incidence of transfusion and identifying patients at higher risk of transfusion are critical to establishing a strategy to decrease blood loss and transfusion rates. The reported incidence of transfusion varies from 3.5 to 18.5% in TKA and from 5.4 to 26.2% in THA, which is negatively associated with hospital procedure volume [5]. Additionally, different blood management strategies also cause the variation of transfusion rates. For orthopedic surgeries, the risk factors for transfusion include advanced age, higher American Society of Anesthesiologists (ASA) grade, lower level of preoperative hemoglobin (Hb), and increased postoperative drainage volume [6].

In this retrospective study, we aim to determine the incidence and risk factors for intraoperative and postoperative allogenic blood transfusion in patients undergoing primary unilateral TKA and THA.

## Methods

This retrospective study included consecutive patients undergoing primary unilateral TKA and THA at our institution between January 2010 and July 2014. For patients who received staged TKAs or THAs for both lower limbs during this period, only the first procedure was included. We excluded patients with coagulation disorders or on anticoagulants before surgery. Patients undergoing autologous blood predonation before surgery were also excluded. This study was approved by our institutional review board (IRB).

All surgeries were performed by four surgeons with standard procedure. The medial parapatellar arthrotomy was used in TKA. A pneumatic tourniquet was applied before skin incision and it was released after cementing the prosthesis. THAs were conducted using modified Hardinge approach. A closed drainage system was employed routinely in TKA and THA, and it was removed within 48 h postoperatively. Tranexamic acid and other hemostatic agents were not used in any of the patients. Recommended thromboprophylaxis, consisting of chemical (rivaroxaban or low-molecular-weight heparin) and mechanical (intermittent pneumatic compression devices) prophylaxis, was used for each patient. All patients underwent a unified rehabilitation program following surgery.

Intraoperatively, the allogenic blood transfusion trigger was a Hb level of less than 9.0 g/dL. Postoperatively, patients were transfused if their level of Hb was less than 8.0 g/dL, or if they presented with obvious anemic symptoms and the level of Hb was less than 9.0 g/dL. All intraoperative and postoperative transfusion events were recorded. We collected patients’ demographic information from medical records, including age, gender, body mass index (BMI), coexisting illnesses, smoking history, and primary diagnosis. The preoperative laboratory values, including Hb, platelet count (PLT), plasma prothrombin time (PT), international normalized ratio (INR), and activated partial thromboplastin time (APTT), were also collected. We obtained the operation time and estimated intraoperative blood loss from operation notes.

Statistical analysis was conducted using STATA version 12.0 (Stata Corp. LP, College Station, TX, USA). Variables, including age, gender, BMI, diabetes, hypertension, malignancy, smoking history, diagnosis, PT, INR, APTT, Hb, PLT, operation time, and intraoperative blood loss, were compared between transfusion group and non-transfusion group. Qualitative variables were analyzed using the chi-square test, and quantitative variables were analyzed using *t* test. Subsequently, factors with *P* value less than 0.05 were introduced into the multivariate logistic regression analysis to identify the independent risk factors for transfusion. A *P* value less than 0.05 was considered statistically significant.

## Results

A total of 1534 patients were enrolled in this study. There were 1067 females and 467 males, with a mean age of 64.8 ± 12.5 years (range 17–93 years). There were 541 patients undergoing primary unilateral TKA and 993 patients undergoing primary unilateral THA. Table 1 shows the baseline characteristics of the study population.

**Table 1:** Patient’s demographic, clinical, and surgical characteristics

Characteristics	Total (*n* = 1534)	TKA (*n* = 541)	THA (*n* = 993)
Gender			
Male (%)	467 (30.4)	97 (17.9)	370 (37.3)
Female (%)	1067 (69.6)	444 (82.1)	623 (62.7)
Age, years (mean ± SD)	64.8 ± 12.5	67.4 ± 7.4	63.4 ± 14.3
BMI, kg/m^2^ (mean ± SD)	24.4 ± 4.0	26.1 ± 3.8	23.4 ± 3.8
Diabetes (%)	184 (12.0)	89 (16.5)	95 (9.6)
Hypertension (%)	548 (35.7)	239 (44.2)	309 (31.1)
Malignance (%)	41 (2.7)	16 (3.0)	25 (2.5)
Smoking (%)	160 (10.4)	32 (5.9)	128 (12.9)
Diagnosis			
OA (%)	643 (41.9)	491 (90.8)	152 (15.3)
RA (%)	64 (4.2)	50 (9.2)	14 (1.4)
Femoral neck fracture (%)	401 (26.1)	–	401 (40.4)
ONFH (%)	255 (16.6)	–	255 (25.7)
DDH (%)	106 (6.9)	–	106 (10.7)
AS (%)	54 (3.5)	–	54 (5.4)
Previous septic arthritis (%)	11 (0.7)	–	11 (1.1)
PT, s (mean ± SD)	11.5 ± 1.0	11.4 ± 0.9	11.6 ± 1.0
INR (mean ± SD)	1.01 ± 0.09	1.00 ± 0.08	1.02 ± 0.09
APTT, s (mean ± SD)	26.9 ± 4.5	25.9 ± 3.8	27.4 ± 4.8
Preoperative Hb level, g/L (mean ± SD)	127.9 ± 14.9	127.3 ± 13.8	128.2 ± 15.5
PLT, 10^9^/L (mean ± SD)	200.4 ± 64.3	206.7 ± 66.9	197.0 ± 62.6
Operation time, min (mean ± SD)	110.3 ± 29.8	124.2 ± 23.1	102.7 ± 30.4
Intraoperative blood loss, mL (mean ± SD)	290.2 ± 168.9	201.3 ± 111.0	338.7 ± 175.2

*TKA* total knee arthroplasty, *THA* total hip arthroplasty, *SD* standard deviation, *BMI* body mass index, *OA* osteoarthritis, *RA* rheumatoid arthritis, *ONFH* osteonecrosis of the femur head, *DDH* developmental dysplasia of the hip, *AS* ankylosing spondylitis, *PT* prothrombin time, *INR* international normalized ratio, *APTT* activated partial thromboplastin time, *Hb* hemoglobin, *PLT* platelet count

Two hundred and 74 patients (17.9%) received allogenic blood transfusion. Among them, 121 patients (7.9%) were transfused during the surgery and 174 patients (11.3%) were transfused after surgery. In the TKA group, the incidence of postoperative transfusion (13.9%, *n* = 75) was higher than that (10.0%, *n* = 99) in the THA group (*P* = 0.022). There was no statistical difference between TKA group and THA group in intraoperative transfusion (7.0% vs. 8.4%, *P* = 0.345) and total transfusion (19.4% vs. 17.0%, *P* = 0.243).

According to the univariate analysis for TKA group, gender (*P* = 0.027), preoperative level of Hb (*P* < 0.001), and intraoperative blood loss (*P* < 0.001) were associated with total transfusion (Table 2). The subsequent multivariate logistic regression analysis showed that preoperative lower level of Hb (*P* < 0.001) and increased amount of intraoperative blood loss (*P* < 0.001) were the independent risk factors for total transfusion in TKA (Table 3). Likewise, we found that prolonged APTT (*P* = 0.035), preoperative lower level of Hb (*P* = 0.007), and increased blood loss (*P* < 0.001) were independent predictors for intraoperative transfusion in TKA. Female (*P* = 0.027) and preoperative lower Hb level (*P* < 0.001) were the independent predictors for postoperative transfusion in TKA.

**Table 2:** Univariate analysis of the risk factors for transfusion in TKA

Variable	Non-transfusion (*n* = 436)	Transfusion (*n* = 105)	*P* value
Female gender (%)	350 (80.3)	94 (89.5)	0.027*
Age, years (mean ± SD)	67.5 (7.2)	66.6 (8.5)	0.264
BMI, kg/m^2^ (mean ± SD)	26.1 (3.7)	26.2 (4.3)	0.762
Diabetes (%)	69 (15.8)	20 (19.1)	0.424
Hypertension (%)	198 (45.4)	41 (39.1)	0.238
Malignance (%)	12 (2.8)	4 (3.8)	0.566
Smoking (%)	28 (6.4)	4 (3.8)	0.308
Diagnosis of RA (%)	36 (8.3)	14 (13.3)	0.107
PT, s (mean ± SD)	11.4 (0.9)	11.5 (0.8)	0.189
INR (mean ± SD)	0.99 (0.08)	1.01 (0.07)	0.192
APTT, s (mean ± SD)	25.8 (3.9)	26.5 (3.7)	0.076
Preoperative Hb level, g/L (mean ± SD)	128.8 (13.2)	121.1 (14.5)	< 0.001*
PLT, 10^9^/L (mean ± SD)	207.3 (65.3)	204.1 (73.5)	0.657
Operation time, min (mean ± SD)	124.1 (22.7)	124.6 (24.5)	0.822
Intraoperative blood loss, mL (mean ± SD)	189.4 (95.5)	250.4 (151.0)	< 0.001*

*SD* standard deviation, *BMI* body mass index, *RA* rheumatoid arthritis, *PT* prothrombin time, *INR* international normalized ratio, *APTT* activated partial thromboplastin time, *Hb* hemoglobin, *PLT* platelet count

*P < 0.05 was considered statistically significant

**Table 3:** Multivariate logistic regression analysis to identify independent risk factors for transfusion in TKA

	OR	95%CI	*P* value
Female	1.545	0.750–3.182	0.238
Preoperative hemoglobin level	0.959	0.942–0.977	< 0.001*
Intraoperative blood loss	1.005	1.003–1.007	< 0.001*

*OR* odds ratio, *CI* confidence interval

*P < 0.05 was considered statistically significant

In THA group, gender (*P* < 0.001), diagnosis (*P* = 0.019), preoperative Hb level (*P* < 0.001), operation time (*P* < 0.001), and intraoperative blood loss (*P* < 0.001) were found to be associated with total transfusion by univariate analysis (Table 4). Subsequently, multivariate logistic regression analysis showed that female (*P* = 0.023), preoperative lower Hb level (*P* < 0.001), prolonged operation time (*P* < 0.001), and increased intraoperative blood loss (*P* < 0.001) were the independent risk factors for total transfusion (Table 5). For intraoperative transfusion in THA, preoperative lower Hb level (*P* < 0.001), prolonged operation time (*P* < 0.001), and increased intraoperative blood loss (*P* < 0.001) were the independent predictors. For postoperative transfusion in THA, female (*P* = 0.037), preoperative lower level of Hb (*P* = 0.007), and increased blood loss (*P* < 0.001) were the independent predictors.

**Table 4:** Univariate analysis of the risk factors for transfusion in THA

Variable	Non-transfusion (*n* = 824)	Transfusion (*n* = 169)	*P* value
Female gender (%)	491 (59.6)	132 (78.1)	< 0.001*
Age, years (mean ± SD)	63.7 (14.1)	61.9 (15.2)	0.142
BMI, kg/m^2^ (mean ± SD)	23.4 (3.7)	23.6 (3.9)	0.518
Diabetes (%)	78 (9.5)	17 (10.1)	0.811
Hypertension (%)	250 (30.3)	59 (34.9)	0.242
Malignance (%)	22 (2.7)	3 (1.8)	0.499
Smoking (%)	107 (13.0)	21 (12.4)	0.843
Diagnosis			0.019*
OA (%)	119 (14.4)	33 (19.5)	
RA (%)	8 (1.0)	6 (3.6)	
Femoral neck fracture (%)	346 (42.0)	55 (32.5)	
ONFH (%)	216 (26.2)	39 (23.1)	
DDH (%)	83 (10.1)	23 (13.6)	
AS (%)	44 (5.3)	10 (5.9)	
Previous septic arthritis (%)	8 (1.0)	3 (1.8)	
PT, s (mean ± SD)	11.6 (1.0)	11.7 (1.1)	0.177
INR (mean ± SD)	1.02 (0.09)	1.03 (0.11)	0.146
APTT, s (mean ± SD)	27.4 (4.9)	27.5 (4.1)	0.698
Preoperative Hb level, g/L (mean ± SD)	130.1 (15.1)	119.1 (14.4)	< 0.001*
PLT, 10^9^/L (mean ± SD)	197.8 (62.5)	193.0 (63.0)	0.361
Operation time, min (mean ± SD)	99.6 (28.0)	117.9 (36.4)	< 0.001*
Intraoperative blood loss, mL (mean ± SD)	314.6 (163.5)	456.0 (183.3)	< 0.001*

*SD* standard deviation, *BMI* body mass index, *OA* osteoarthritis, *RA* rheumatoid arthritis, *ONFH* osteonecrosis of the femur head, *DDH* developmental dysplasia of the hip, *AS* ankylosing spondylitis, *PT* prothrombin time, *INR* international normalized ratio, *APTT* activated partial thromboplastin time, *Hb* hemoglobin, *PLT* platelet count

*P < 0.05 was considered statistically significant

**Table 5:** Multivariate logistic regression analysis to identify independent risk factors for transfusion in THA

	OR	95%CI	*P* value
Female	1.694	1.074–2.674	0.023*
Diagnosis	1.041	0.917–1.182	0.533
Preoperative hemoglobin level	0.948	0.934–0.961	< 0.001*
Operation time	1.015	1.009–1.021	< 0.001*
Intraoperative blood loss	1.004	1.003–1.005	< 0.001*

*OR* odds ratio, *CI* confidence interval

*P < 0.05 was considered statistically significant

## Discussion

The incidence of transfusion in TJA, TKA, and THA was 17.9%, 19.4%, and 17.0%, respectively. In TKA, preoperative lower level of Hb and increased amount of intraoperative blood loss were significantly associated with transfusion. In THA, female, preoperative lower Hb level, prolonged operation time, and increased intraoperative blood loss were the independent risk factors for transfusion. Preoperative optimization and preventive measures for these risk factors may be able to decrease transfusion rates.

Total joint arthroplasty is usually associated with excessive blood loss, which may cause a mean Hb decrease of 3.7 g/dL [7] and requirements for allogenic transfusion. Because of different patient and hospital characteristics, the reported incidence of transfusion varies widely from 2.5 to 35.3% in TKA and from 14 to 29.8% in THA [5, 8–12]. Studies about the trends in allogenic transfusion reveal a decline in TKA and an increase in THA [9, 13]. Transfusion is found to be dose-dependently associated with surgical site infection following TJA [3, 14]. It also increases the risk of postoperative cardiac arrhythmia, confusion, and urinary catheterization [15]. Patients receiving transfusion during TJA are prone to higher cost, prolonged length of stay, and discharge to short-term care [13]. Moreover, transfusion appears to increase the rates of in-hospital and 1-year postoperative mortality in TJA [15, 16].

Preoperative Hb level is considered a significant predictor for transfusion following TJA [11, 17, 18], which is consistent with the present study. Patients with a preoperative Hb level less than 13 g/dL are at a fourfold higher risk of having transfusion compared to those with Hb level of > 13 g/dL [18]. Bleeding and clotting disorders are also independent risk factors for transfusion [14], and we excluded patients with these comorbidities from our study. According to previous studies, female and older age have proven to be associated with transfusion in TKA and THA [8, 19, 20]. In our study, female is found to be the independent risk factor for postoperative transfusion but not total or intraoperative transfusion in TKA; it is the independent risk factor for total and postoperative transfusion but not intraoperative transfusion in THA. We did not find the relationship between age and transfusion rates.

There are several limitations in our study. First, this study has all limitations inherent to retrospective observational studies, which should be noted when interpreting the results. Second, we only recorded the events of transfusion during hospitalization. Some patients may receive transfusion after discharge to another hospital for short-term inpatient care. Third, postoperative parameters (drainage volume, anticoagulants use, etc.) and other confounders (ASA grade, comorbidity index, etc.) may also influence transfusion rates, but we did not include these factors into the analysis. Fourth, the value of intraoperative blood loss was estimated by the volume of blood in the suction canister plus the amount of blood in the dry sponges, which may cause the inaccuracy of the data. It also should be noted that the prevalence of transfusion in this study may be higher, because we did not optimize preoperative Hb concentration or use tranexamic acid during the research period.

Given the high incidence of transfusion in TJA, further research to decrease blood loss and optimize blood management is warranted. For patients with identified risk factors, perioperative interventions should be employed to reduce the transfusion rates.

## Data Availability

The data used to support the findings of this study are available from the corresponding author upon request.

## Abbreviations

APTT: Activated partial thromboplastin time
ASA: American Society of Anesthesiologists
BMI: Body mass index
Hb: Hemoglobin
INR: International normalized ratio
IRB: Institutional review board
PLT: Platelet count
PT: Plasma prothrombin time
THA: Total hip arthroplasty
TJA: Total joint arthroplasty
TKA: Total knee arthroplasty
